# Supplementary figures and images for: A single epidermal stem cell strategy for safe ex vivo gene therapy
Source: EMBO Mol Med. 2015 Feb 27;7(4):380–93. doi: 10.15252/emmm.201404353 (PMC4403041; doi:10.15252/emmm.201404353)

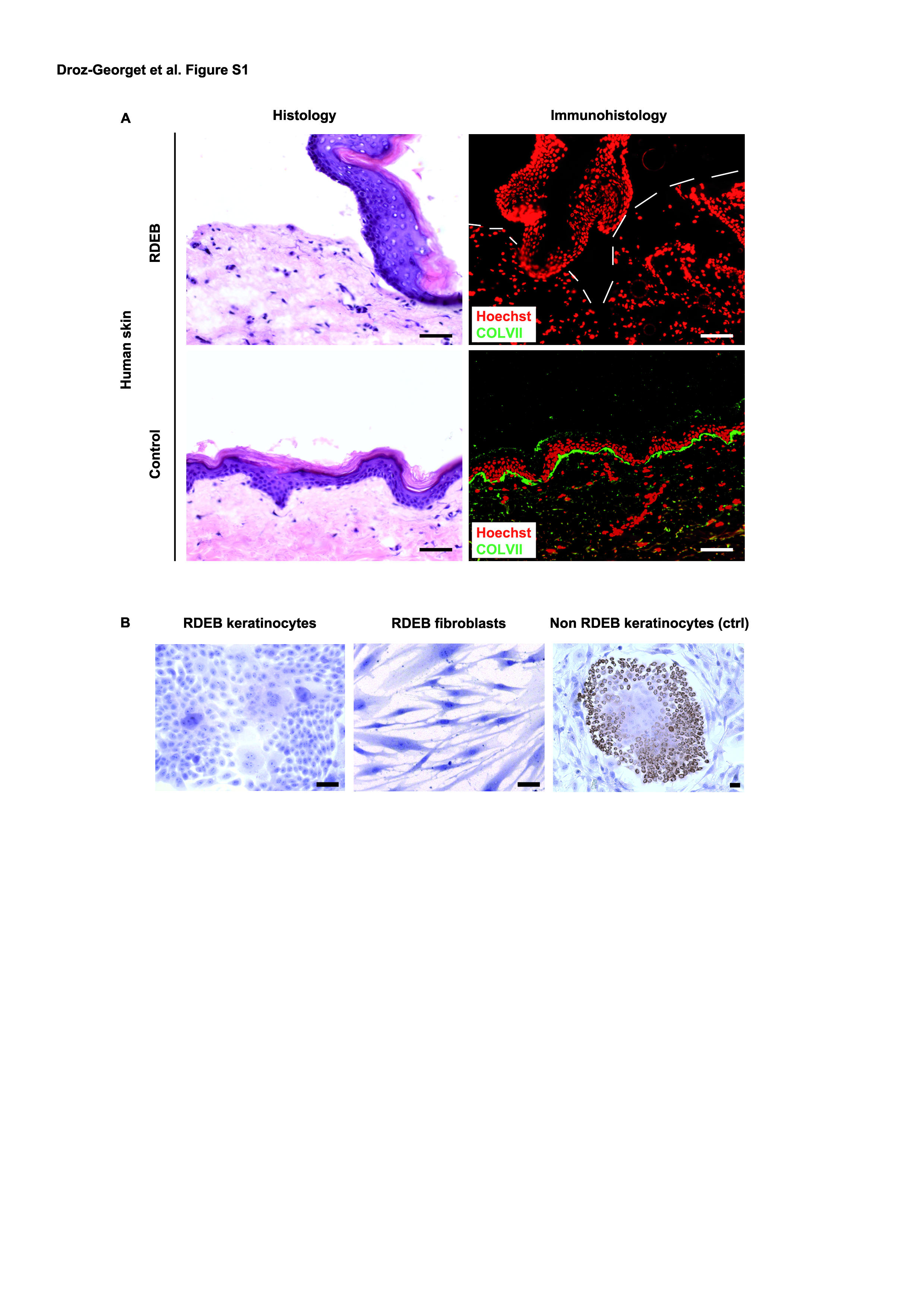

Supplement: Supplementary file 1 — Supplementary Figure S1 [file emmm0007-0380-sd1.tif]

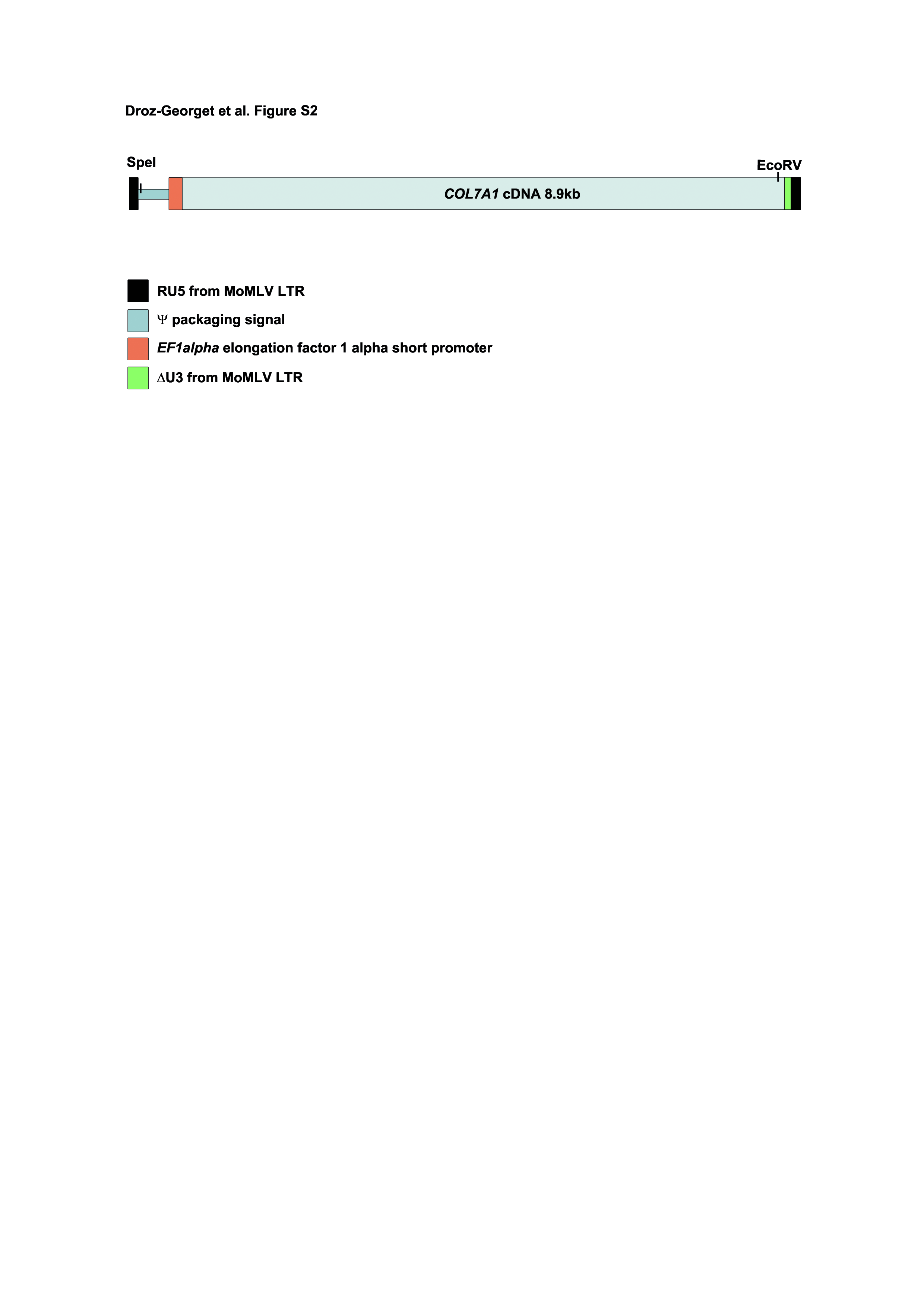

Supplement: Supplementary file 2 — Supplementary Figure S2 [file emmm0007-0380-sd2.tif]

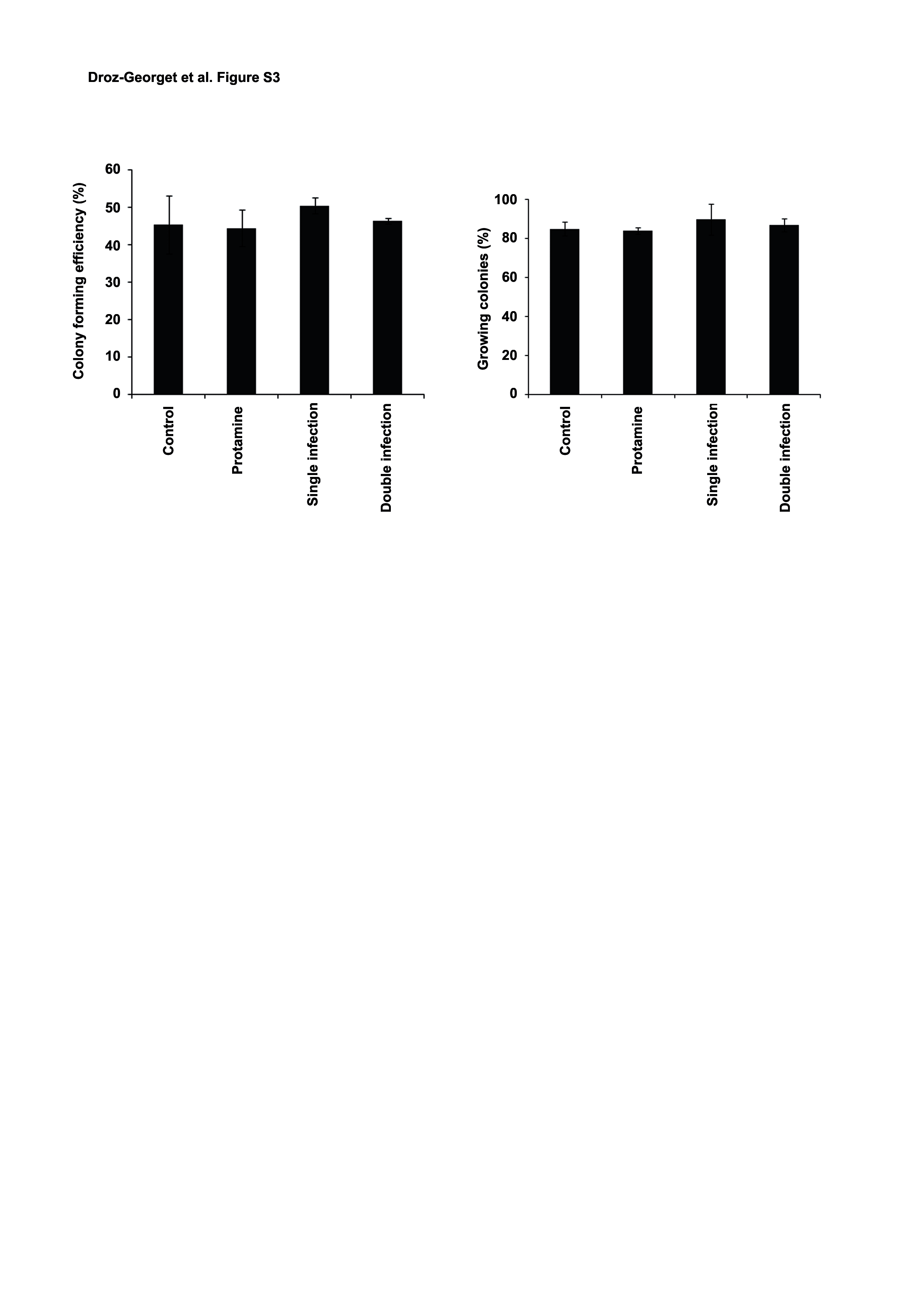

Supplement: Supplementary file 3 — Supplementary Figure S3 [file emmm0007-0380-sd3.tif]

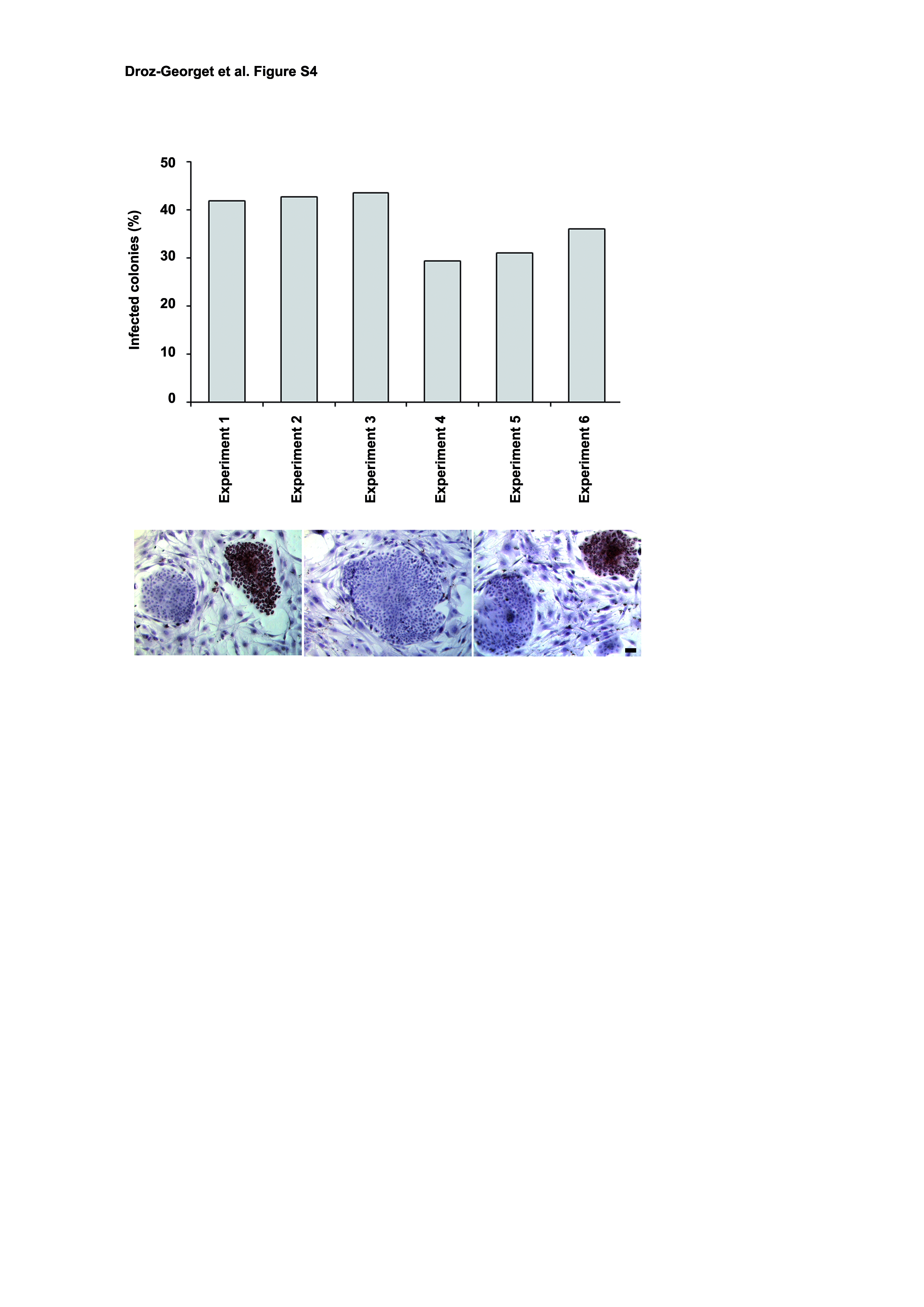

Supplement: Supplementary file 4 — Supplementary Figure S4 [file emmm0007-0380-sd4.tif]

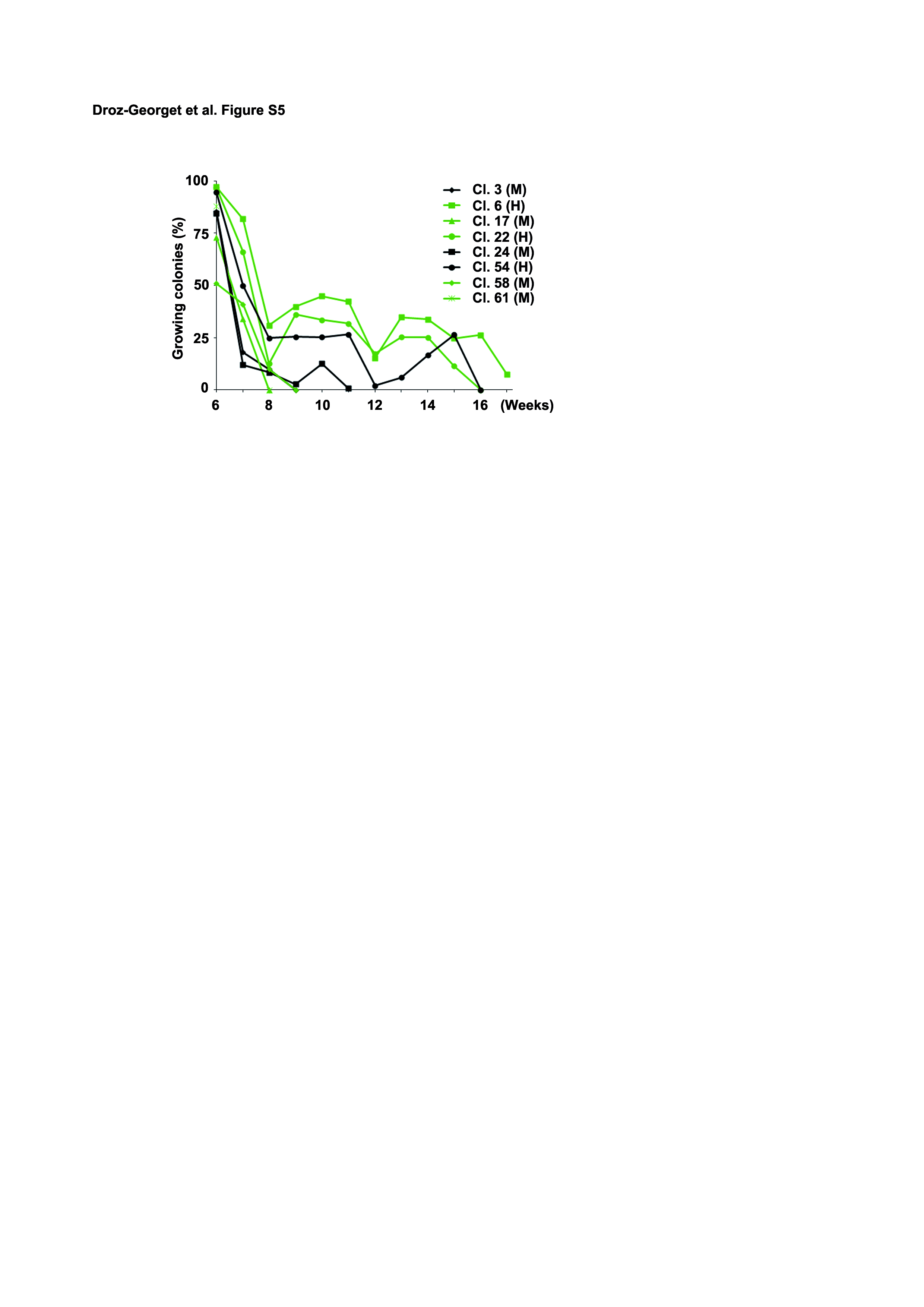

Supplement: Supplementary file 5 — Supplementary Figure S5 [file emmm0007-0380-sd5.tif]

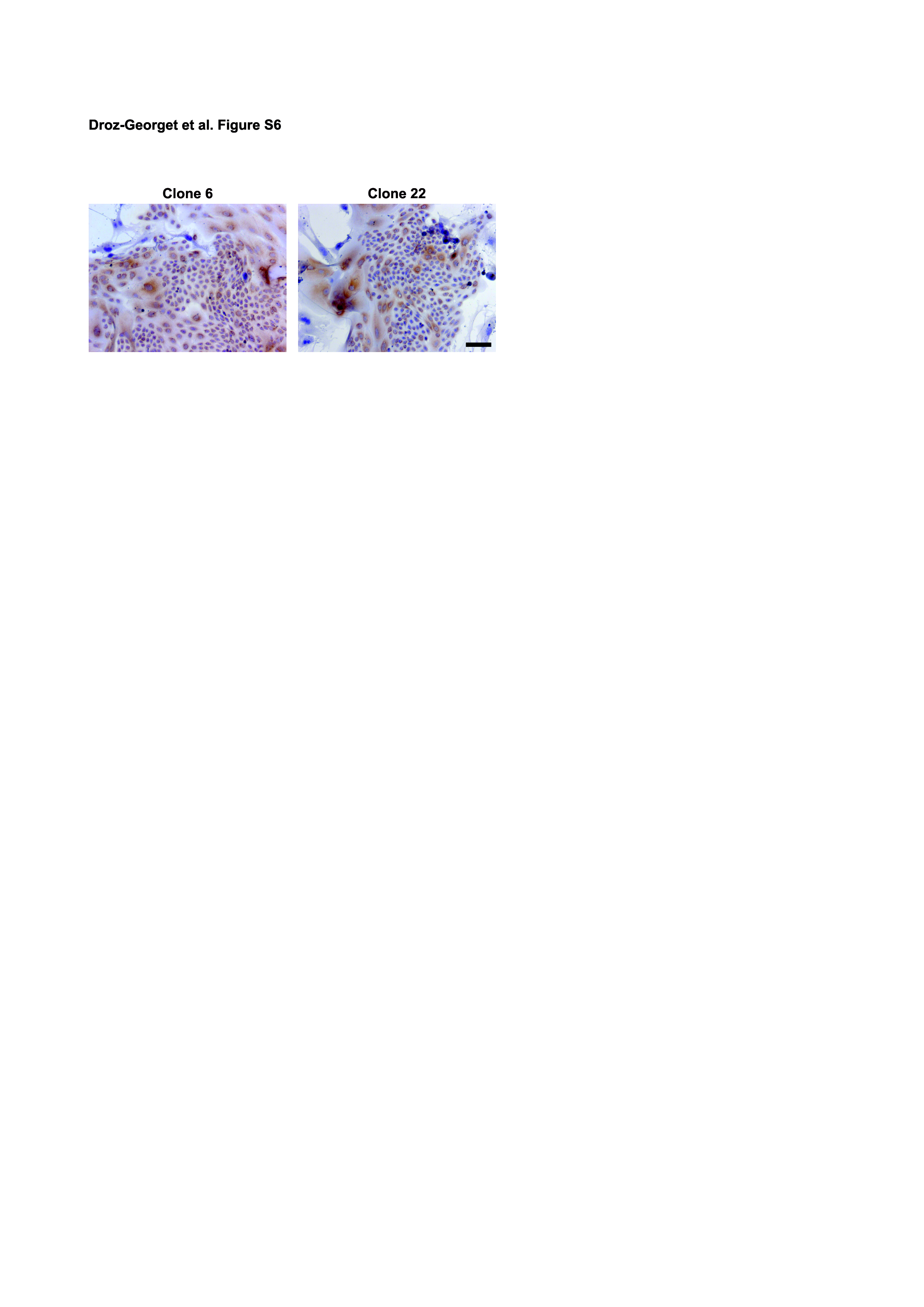

Supplement: Supplementary file 6 — Supplementary Figure S6 [file emmm0007-0380-sd6.tif]

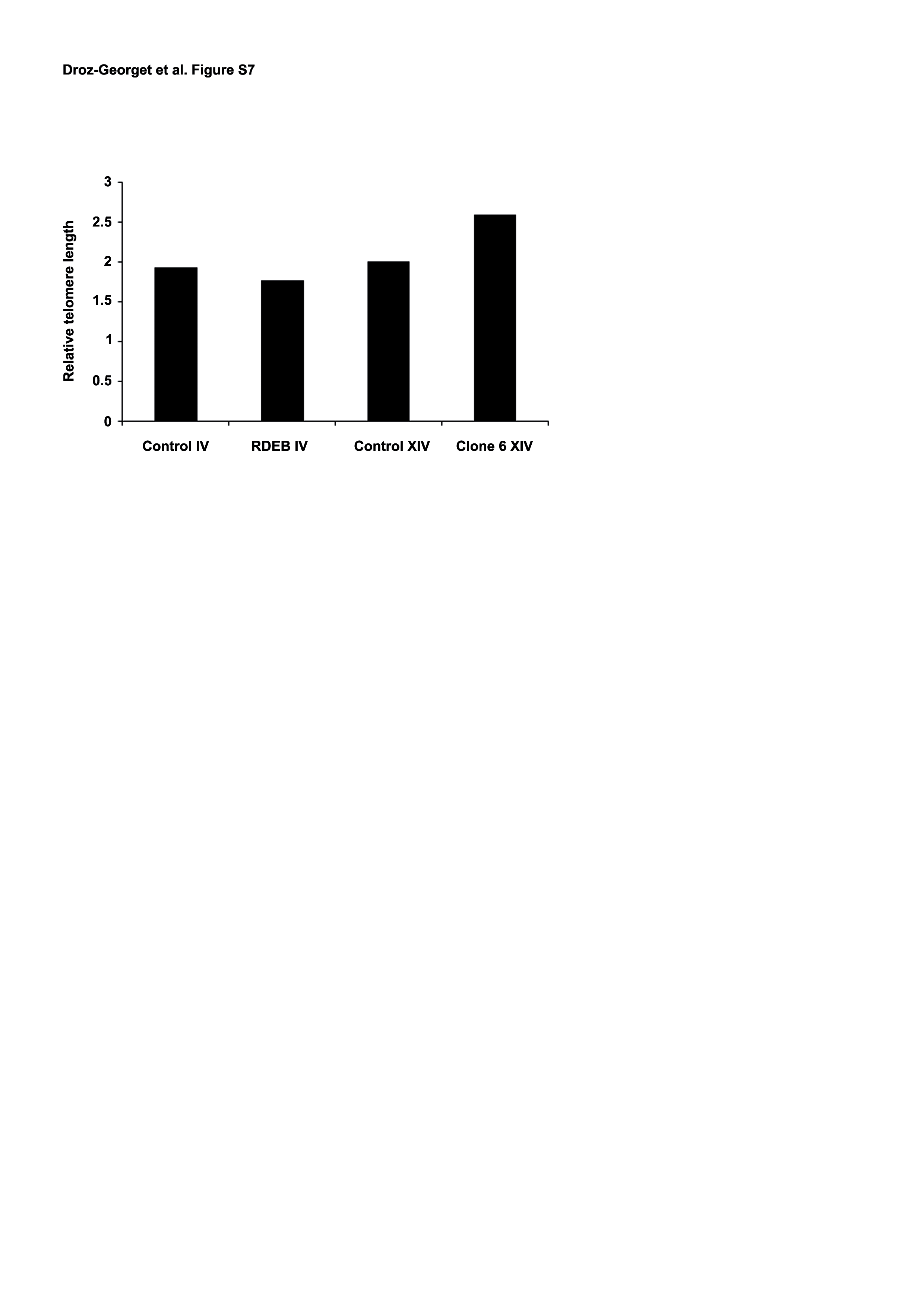

Supplement: Supplementary file 7 — Supplementary Figure S7 [file emmm0007-0380-sd7.tif]

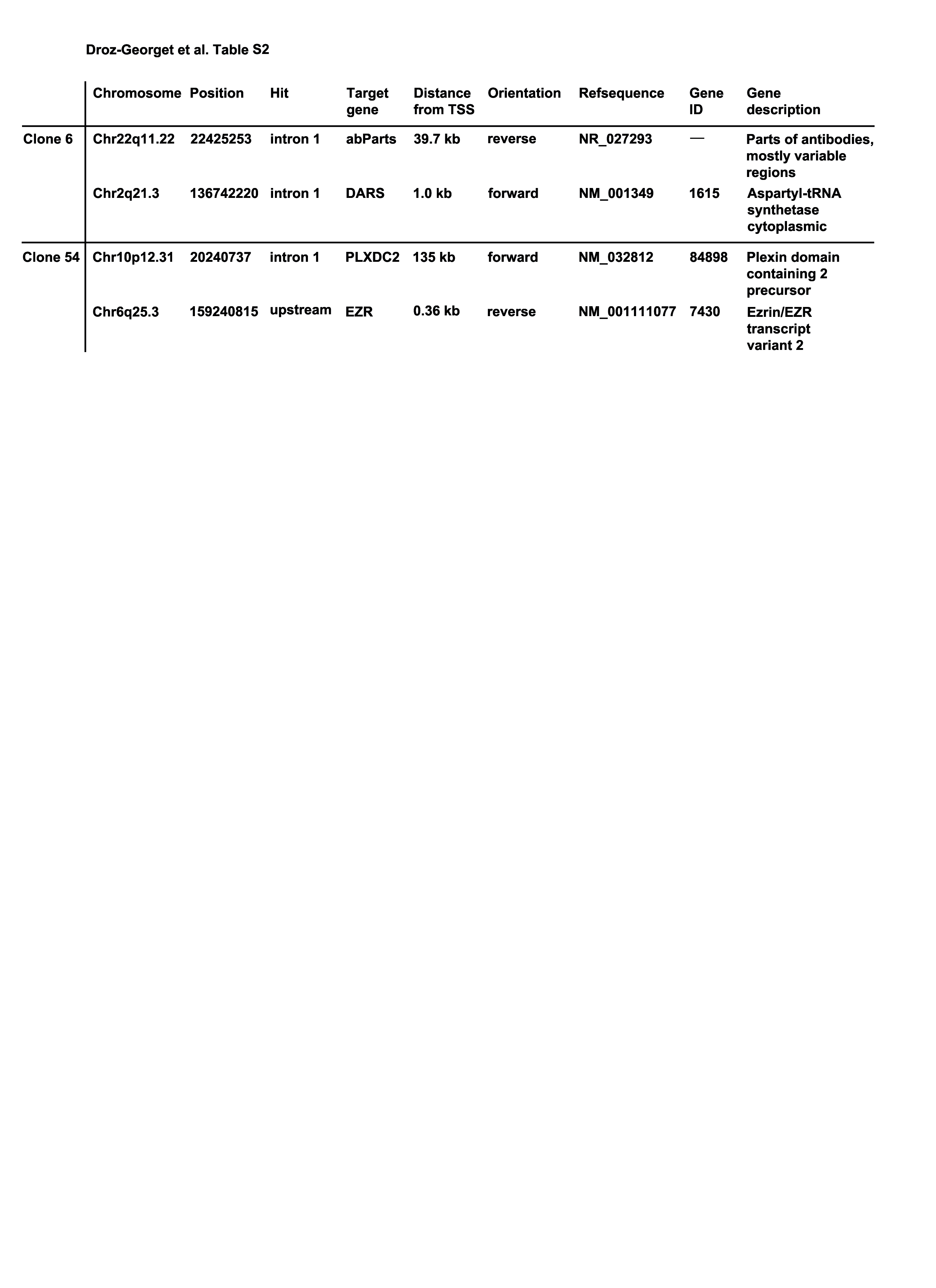

Supplement: Supplementary file 9 — Supplementary Table S2 [file emmm0007-0380-sd9.tif]
