# Supplementary material for: A single epidermal stem cell strategy for safe ex vivo gene therapy
Source: EMBO Mol Med. 2015 Feb 27;7(4):380–93. doi: 10.15252/emmm.201404353 (PMC4403041; doi:10.15252/emmm.201404353)
Supplement: Supplementary file 10 — Supplementary Information [file emmm0007-0380-sd10.doc]

Supplementary Information

Materials and Methods

1. Cell culture p 2
2. Clonal analysis and serial transfer p 2
3. Vector production and titration p 3

###### Retroviral infection p 3

###### Transplantation of cultured epithelium p 3-4

1. Immunodetection and histology p 4
2. Electron microscopy p 5
3. Western blotting p 5-6

##### Karyotype and fluorescence in situ hybridisation p 6

1. Tumorigenic and dissemination assays p 6-7
2. Quantitative reverse transcriptase PCR p 7
3. Relative telomere length analysis p 7
4. Southern blotting p 8
5. Ligation-mediated PCR p 8

Supplementary legends

**1. Cell culture**

Human keratinocytes or fibroblasts were isolated from a biopsy obtained from the wrist of a 4 year-old RDEB patient (Hilal *et al*, 1993), from the foreskin of a newborn (YF29), and from the groin of a 42 year-old female (OR-CA). Tissues were dissociated in 0.05% trypsin – 0.1% EDTA at 37°C. Keratinocytes were then cultured onto a feeder layer of lethally irradiated 3T3-J2 cells (Rheinwald & Green, 1975) in medium (cFAD) consisting in a 3:1 mixture of Dulbecco’s Modified Eagle’s Medium (DMEM) and Ham’s F-12 medium (Gibco) containing 10% Foetal Calf Serum (FCS) (Hyclone) and supplemented as described (Rochat *et al*, 1994, Ronfard *et al*, 2000). 10ng/ml recombinant human epidermal growth factor (rhEGF) (QED Biosciences) was added at the first feeding. For feeder layer, 3T3-J2 cells were lethally irradiated (60 Gy) and seeded at a density of 2.5x10^4^ cells/cm^2^. 3T3-J2 cells were grown in DMEM supplemented with 10% Bovine Serum (BS) (Hyclone). RDEB fibroblasts were cultured in DMEM supplemented with 10% FCS. The squamous cell carcinoma cell line SCC-13 (Rheinwald *et al*, 1981) was cultivated on a feeder layer of irradiated 3T3-J2 cells in cFAD supplemented with 10% FCS. The Flp293A-E1aColVII1 was cultured in DMEM supplemented with 10% heat inactivated FCS. Human keratinocytes were frozen in 10% glycerol in cFAD medium, human fibroblasts in DMEM supplemented with 10% DMSO (dimethylsulfoxide) and 20% FCS, whereas 3T3-J2 cells were frozen in DMEM supplemented with 10% DMSO and 20% BS. All cultures were incubated at 37°C in a 10% CO_2_ atmosphere and subcultured once a week.

**2. Clonal analysis and serial transfer**

Single cells were isolated as described (Barrandon & Green, 1985). Briefly, 150 individual cells trypsinised from a mass of infected cells were aspirated into a Pasteur pipette under a Zeiss Axiovert inverted microscope using a 10x objective and immediately inoculated into a 35mm size Petri dish already containing lethally irradiated 3T3-J2 cells. Cultures were fed every 3-4 days with cFAD medium. Clonal types were determined as described (Barrandon & Green, 1987). Selected keratinocyte clones were subcultured once a week or frozen as described above. For determination of colony-forming efficiency, 100 cells were seeded in a 60 or 100mm size Petri dish and cultured for 12 days, after which cultures were fixed in 3.7% formaldehyde (Sigma), stained with 1% Rhodamine B and colonies were counted under a dissecting microscope (Leica). The growth potential of keratinocytes was evaluated by serial transfer as described in Rochat *et al*, 1994. Briefly, mass culture and individual clones were passaged until no colony formed. The plating efficiency and the percentage of growing colonies for each passage were used to calculate the population doubling, the generation number and the total progeny.

**3. Vector production and titration**

The Flp293A-EIaColVII1 producer clone was generated by Genethon, Evry, France as described (Schucht *et al*, 2006). Briefly, the pBullet-MinSin3-NS-EF1alpha-COL7A1 retroviral construct was introduced into a HEK293-based retroviral producer cell line (Flp293A) by the Flp recombinase technology. The construct contains a self-inactivating (SIN) LTR, the complete cDNA sequence of *COL7A1* under the control of a minimal promoter of human elongation factor 1 alpha. Culture medium containing retroviruses was collected at 24, 48 and 72 hours, pooled, 0.45μm filtered, aliquoted and stored at –80°C. Viral titres were determined by 2 hours transduction of HCT116 cells with serial dilutions of the vector preparations in the presence of 6μg/ml polybrene (Sigma). 72h later, genomic DNA from transduced cells was extracted and the infectious particles titre (IP/ml) was determined by Taqman quantitative PCR as described (Titeux *et al*, 2010).

**4. Retroviral infection**

5x10^4^ irradiated 3T3-J2 cells were plated on 35mm size Petri dishes in cFAD medium. One and a half hours later, 2x10^4^ keratinocytes from early passage were seeded onto the 3T3-J2. Infection was performed 16-24 hours later in 500μl infection medium with viral supernatant corresponding to a theoretical MOI of 10 for 5 hours at 37°C in a 10% CO_2_ incubator. 500μl medium was added and cells kept overnight. The medium used for the infection process was cFAD medium prepared with 10% heat inactivated FCS (Hyclone) in the presence of rEGF (QED) and protamin (5μg/ml) (Valeant Pharmaceuticals). The infection process was repeated 24 hours after the first round. The infection efficiency was determined by immunodetection against COLVII one passage after infection on colonies cultured at low-density (200 cells seeded per 35 mm size Petri dish).

**5. Transplantation of cultured epithelium**

Cells were cultured on a fibrin-based matrix as previously described (Larcher *et al*, 2007). Briefly, a 40ml aliquot of frozen human plasma from blood donors was thawed and centrifuged at 3300 rcf (relative centrifugal force) for 30 minutes at 4°C. The pellet was solubilised in 6ml calcium and magnesium free Dulbecco’s phosphate buffered saline (DPBS Gibco) for 15 minutes at 37°C. Each millilitre was then mixed with 2ml of DMEM supplemented with 10% FCS and 150IU/ml aprotinin (Trasylol, Bayer), and 8x10^4^/ml uncorrected RDEB autologous fibroblasts (passage 11). Human thrombin (5U Tissucol kit, Baxter) was then added to favour the formation of the fibrin gel. 2ml of the mix was then poured in each well of a 12 well plate and incubated overnight in medium. 10^5^ keratinocytes were then seeded on top of the fibrin gel and grown to confluence in culture medium supplemented with 150IU/ml aprotinin (Trasylol, Bayer). A skin flap was made onto the back of 8-10 weeks-old Fox-Chase SCID mice (Charles River Laboratories) to expose the muscular fascia. The cultured epithelia were then detached from the culture vessels and transplanted onto the mouse, the fibrin matrix facing the thoracic muscular fascia. The grafts were then stitched to the thoracic wall, covered with Urgotul (Laboratoires Urgo, France) and a small piece of silicone sheet (0.13 mm thickness) (Dow corning). The flap was then folded back in its original place and stitched. After 21 days, the flap was excised and the graft was air exposed. Mice were handled according to the Canton de Vaud veterinarian guidelines (authorisation 2033). Grafts were harvested at different time points and processed for histology, immunocytochemistry or electron microscopy.

**6. Immunodetection and histology**

Cultured cells were fixed in ice-cold methanol/acetone (v:v) for 20 minutes, blocked with 5% FCS (Hyclone) in Phosphate Buffered Saline (PBS) (Sigma) and immunostained according to standard immunofluorescence or DAB (3,3'-diaminobenzidine) protocols. The primary antibody was a mouse anti-human COLVII (1:1000; clone LH7.2. Sigma) and the secondary antibody was an Alexa Fluor 488 anti-mouse IgG (1:200; Invitrogen). Nuclei were counterstained with 10µM Hoechst 33342 (Fluka). Preparations were then mounted using a fluorescence mounting medium (Dako) and visualised using an inverted fluorescence microscope (Axiovert, Zeiss). Photographs were taken using a Zeiss AxioCam camera (AF488 signal in green and Hoechst signal in blue). DAB (3,3'-diaminobenzidine) cytochemistry was performed according to standard protocols. Briefly, cells were treated as described above and detection was made with the ENVISION mouse kit (K4006 DAKO) according to the manufacturers’ instructions. Plates were counterstained in Harris Haematoxylin (Sigma).

Transplants were biopsied using a 2mm diameter punch (Stiefel). Biopsies were fixed for 2 hours in 4% paraformaldehyde (MERCK), washed in PBS, incubated overnight in 30% cold sucrose (Sigma), embedded in OCT compound (Tissue-Tek) and frozen (-80°C). 7μm sections were then obtained using a Leica CM 3000 cryostat and treated with BS (Hyclone) supplemented with 0.5% triton. Sections were immunostained using standard protocols for human HLA class I (1:500 - SM2012P Acris), human COLVII (1:500 - LH7.2 antibody - Sigma) or human Ki67 (1:500 - Becton Dickinson 550609). Secondary antibodies were Alexa Fluor 488 conjugated anti-rat or anti-rabbit IgG and Alexa Fluor 568 conjugated anti-mouse IgG (1:200) (Invitrogen) respectively. Nuclei were counterstained as described above. Sections were then mounted in fluorescent mounting medium (Dako) and examined as described above. Skin samples from RDEB patient and mastectomy were obtained after surgery and processed as above, excepted that fluorescent signals, pictured in black and white were false coloured in green (AF568 signal) and red (Hoechst signal) .

Histological analyses were performed on section parallel to immunostaining. Sections were progressively rehydrated, stained with haematoxylin and eosin (Sigma), dehydrated in alcohol-baths, and mounted in Eukitt (Kindler).

**7. Electron microscopy**

Skin biopsies were immersed for 2 hours in 2.5% glutaraldehyde and 2% paraformaldehyde in 0.1M phosphate buffer (PB). 50µm sections were cut with a vibratome (Leica VT100), fixed for a further 2 hours, washed with 0.1M cacodylate buffer, postfixed 40 minutes in 1% osmium tetroxide containing 3% potassium ferrocyanide, and then in osmium tetroxide alone. Samples were then stained 30 minutes in 1% uranyl acetate, dehydrated through increasing concentrations of alcohol and embedded in Durcupan ACM (Fluka) resin in a 60°C oven. Sections 1µm thick were cut with a glass knife and 50nm thick sections were cut using a diamond knife (Diatome) and an ultramicrotome (Leica UCT). Sections were contrasted with uranyl acetate and lead citrate before being imaged with a Phillips CM10 transmission electron microscope at a filament voltage of 80kV. Images were collected using a CCD camera (Morada, SIS). For immuno-electron microscopy, punches were fixed with 0.2% glutaraldehyde and 2% paraformaldehyde in 0.1M PB for 2 hours at 4°C. They were vibratome (Leica VT100) sectioned into 50µm slices, cryoprotected in 20% DMSO/2% glycerol in 0.1M PB 15 minutes and freeze thawed twice in liquid nitrogen. After washing three times in 0.1M PB, sections were pre-treated in 0.05M glycine in PBS for 10 minutes, blocked in 0.1% BSA (Aurion) and incubated overnight with anti-COLVII antibody (pAb 234192, Calbiochem). After washing, samples were exposed to the gold labelled secondary antibody (1:100, goat anti-rabbit (F)ab fragment from Ultra Small ImmunoGold, Aurion) 4 hours, washed in 0.15M HEPES buffer pH8. Ultra small gold particles were silver enhanced using pre-prepared solutions (Aurion R-Gent SE-EM, Aurion) and left in the dark 50 minutes. Sections were washed in HEPES buffer, in 0.1M cacodylate buffer and postfixed in 1% osmium tetroxide in 0.1M cacodylate buffer, dehydrated in alcohol and then embedded in resin as above.

**8. Western blotting**

To detect endogenous COLVII production, keratinocytes were treated with 20ng/ml Transforming Growth Factor β2 (TGFβ2) (Abcam ab 629) and 50ng/ml ascorbic acid (A-4034 Sigma) for 24 hours in serum free cFAD medium. Supernatants were incubated in Complete Protease Inhibitor Cocktail (Roche) and concentrated with Centricon plus-20 tubes (Millipore). 70μg of proteins were loaded on a 4-12% NuPAGE gradient gel (Invitrogen) and run in MOPS (Fluka)/SDS running buffer according to the manufacturer’s instructions. The gel was then transferred onto a nitrocellulose membrane (PROTRAN, Whatman) in a 1% methanol (MERCK) Tris-glycine based transfer buffer. Immunoblots were performed using anti-COLVII antibody at a 1:2000 dilution (pAb 234192, Calbiochem) and anti-MMP2 at a 1:500 dilution (ab51127, Abcam). Secondary antibodies were goat anti-rabbit IgG antibodies (1:5000) conjugated to HRP (Jackson Immunoresearch). Cell extracts were prepared according to the localisation of the protein of interest using the ProteoExtract Subcellular Proteome Extraction kit (Calbiochem). Whole cell extracts were obtained with RIPA buffer (150mM NaCl, 50mM Tris-HCl, 0.1%SDS, 0.5% Na deoxycholate, 1% Triton X-100). 12μg of proteins were loaded onto 6 to 12% polyacrylamid gel. Loading controls were performed according to the type of cell extract (Histone H3 for nuclear, GAPDH for cytoplasmic and Tubulin for whole extracts). Antibodies used: to detect pRb (554136 BD Pharmingen), p53 (Ab-12 DO-7 Millipore), Ras (16117 Thermo Scientific), p16 (551153 BD Pharmingen), Histone H3 (4499 Cell Signaling), α-Tubulin (T5168 Sigma), GAPDH (ab8245 Abcam). The chemiluminescent detection system was from Pierce.

##### 9. Karyotype and fluorescence in situ hybridisation

Cells were treated for 6 hours with 0.2μg/ml colcemid (1512-012, Gibco), trypsinised and incubated 10 minutes in 75mM KCl. Chromosomes were fixed in cold 3:1 methanol/acetic acid and sent to ChromBios, Germany for chromosome counting and karyogram determination. Briefly, slides were stained with DAPI and mounted in an antifading medium. Metaphase chromosomes were analysed with a Zeiss Axioplan II microscope equipped with a b/w CCD camera. Image capture was done with SmartCapture software (Digital Scientific). Images were printed and counts made manually on the printout. For karyotyping, the image processing software Quips (Vysis) for reverse DAPI banding was used. For fluorescent *in situ* hybridisation (FISH) analysis, spreads of metaphase chromosomes were obtained according to standard procedures with a supplementary pre-fixation step (90% H_2_O, 5% acetic acid and 5% methanol). 500ng of isolated *COL7A1* cDNA was labeled by nick-translation with Spectrum Red according to the manufacturer's protocol. The probe was cohybridised either with a probe on 22q11.2 or on the centromere of chromosomes 2, 3 or 11 (respectively BAC clone CTA-154H4, D2Z1, D3Z1 and D11Z1, kindly provided by Dr. M. Rocchi, University of Bari). FISH was performed according to protocol as described (Pinkel *et al*, 1988).

**10. Tumorigenic and dissemination assays**

Transduced keratinocytes or SCC-13 cells were resuspended at a concentration of 10^7^ cells per ml medium. 100μl of the cell suspension was then inoculated subcutaneously into the ventral flanks of 7 to 9 weeks old athymic Swiss Nu^-/-^ mice (Charles Rivers Laboratories) with a 21-gauge needle. 4-10 injections were made for each cell type. Tumour formation was monitored twice a week and their diameter measured. Corresponding volumes were calculated with the 4/3πr^3^ formula. The experiments were stopped when tumours initiated by SSC-13 cells reached 1cm diameter. For dissemination experiments, internal organs of SCID mice transplanted with recombinant COLVII keratinocytes were harvested at the termination of the experiment. DNA was extracted using the QIAamp DNA mini kit (Qiagen) according to the manufacturer’s instructions. 100ng of DNA were submitted to PCR (BioConcept) amplification with GoTaq PCR reagent kit (Promega) for 40 cycles with *COL7A1* primers. Primers for *COL7A1* were: forward, 5’-CCCTGAGGAGCTGAAG-3’; reverse, 5’-CTGTACTCTCAAGGATTGG-3’; *β-actin* primers were: forward, 5’-TCATGTTTGAGACCTTCAACACCC; reverse, 5’-GTACTTGCGCTCAGGAGGAG-3’. PCR products were resolved by agarose gel electrophoresis, stained with ethidium bromide and visualised under ultraviolet light. Fasteris (Switzerland) sequenced the PCR products.

**11. Quantitative reverse transcriptase PCR**

Cells were lysed in TRIzol (Invitrogen) and total RNAs extracted using the RNA extraction kit (Qiagen) according to the manufacturer’s instructions and as described (Bonfanti *et al*, 2010). Reverse transcription was performed using the Superscript III reverse transcription kit (Invitrogen). Random primers were used at a concentration of 3μg/ml, the dNTPs mix at 0.5mM (Qiagen) and the RNase inhibitor at 2U/μl (Promega). Total cDNAs were diluted ten folds in nuclease-free H_2_O (Roche) and 2μl of the mix was amplified with the Light-Cycler FastStart DNA Master SYBR Green I kit (Roche Diagnostics) in a 12.5μl volume per capillary. The reaction contained 1.15μl FastStart reaction mix, 4mM MgCl_2_ and 0.5μM of each specific primer. Primers were designed with the Light-Cycler Probe Design 3 program (Roche) for an annealing temperature of 60°C and ordered to Microsynth. Quantitative PCR reactions were performed as followed: 95°C 15 minutes, 45 cycles of 95°C 10 seconds, 55°C 5 seconds and 72°C 6 seconds. The program for *DARS* was the following: 95°C 15 minutes, 40 cycles of 95°C 10 seconds, 58°C 5 seconds and 72°C 9 seconds. For data analysis, the housekeeping TATA box binding protein gene (*TBP*) was used as internal control. Primers for *COL7A1* were: forward 5’-CCCTGAGGAGCTGAAG-3’, reverse 5’-CTGTACTCTCAAGGATTGG-3’; for *DARS:* forward 5’-AGTTCCCATGTGAGCCATTC-3’, reverse 5’-TTTGGTGTGCTCAGAATCGTC-3’ and for *TBP*: forward 5’-ATAATCCCAAGCGGTT-3’, reverse 5’-ACTTTAGCACCTGTTAATAC-3’. All PCR products were sequenced (Fasteris, Switzerland).

**12. Relative telomere length analysis**

Genomic DNA was extracted with the Wizard® Genomic DNA purification kit (Promega) according to the manufacturer’s protocol. Relative telomere length was assessed using a QPCR based method (Cawthon, 2002). The relative telomere length was measured by calculating the difference between the average Ct values of the Telomere and the HBG PCR products (T/S ratio). Briefly, 15 ng of genomic DNA was amplified with the Power SYBR® Green Master Mix (Life Technologies) with either the primer pair for Telomere or HBG in two separate 96 well-plates. The primer sequences and final concentrations were as follows: *TEL*-1: CGGTTTGTTTGGGTTTGGGTTTGGGTTTGGGTTTGGGTT (300nM); *TEL*-2: GGCTTGCCTTACCCTTACCCTTACCCTTACCCTTACCCT (600nM); *HBG*-1: TGTGCTGGCCCATCACTTTG (450nM); *HBG*-2: ACCAGCCACCACTTTCTGATAGG (450nM). The PCR cycles were as follows: for Telomere, one step at 95°C for 10 minutes, followed by 40 cycles of 95°C for 15 seconds and 54°C for 2 minutes; for HBG, one step at 95°C for 10 minutes, followed by 40 cycles of 95°C for 15 seconds and 58°C for 1 minute. All reactions were run in quadruplicates.

**13. Southern blotting**

Genomic DNA was extracted with QiAmp DNA kit (Qiagen) according to the manufacturer’s instruction. 10µg of DNA was codigested with SpeI/EcoRV HF (New England Biolabs) and loaded on a 0.8% agarose (Promega) gel. The gel was submitted to depurination under UV, denaturation in 0.2M NaOH, 0.6M NaCl and neutralisation in 1M Tris-HCl pH7.5, 1.5M NaCl. The DNA was transferred onto Hybond XL (GE Healthcare Amersham) membrane in 10x saline-sodium citrate buffer (SSC) by capillarity. The DNA was fixed onto the membrane with UV (Stratalinker, Stratagene), the membrane washed in 0.1% SSC, 0.5% SDS and prehybridised in 5xSSC Phosphate buffer (SSCP), 5xDenhardt (Sigma), 50% formamid (Sigma). Hybridisation was performed at 42°C with ^32^P dCTP (Perkin Elmer) radiolabelled probe in 6xSSC, 10% dextran-sulfate (Sigma), 50% formamid (Sigma), 0.5% SDS, 1x Denhardt’s (Sigma) and 1xS256 solution (composed of 1.6mg polyA (Sigma), 1.6 mg polyC (Sigma), 40mg yeast t-RNA (Sigma), 10mg salmon sperm DNA (Sigma) and 0.9 mg *E. coli* DNA (Sigma)). The probe was labelled using random priming kit (Roche) and purified by spin column (GE Healthcare Amersham). The template of the probe was obtained from PCR amplification of pTOPO*COL7A1* with specific primers ordered from Microsynth: forward 5’-TGATTGCCCTCTACGC-3’, reverse 5’-CGTTGTATCTGACACC-3’. The membrane was washed at room temperature in 2xSSC, 0.1% SDS and 60°C in 0.2xSSC, 0.1% SDS and placed against the screen of a phospho-imager FLA-300 (Fujifilm).

**14. Ligation-mediated PCR**

To remove 3T3-J2 contaminants that could impair the determination of the proviral integration sites, keratinocytes were subcultured twice without feeder layer in KBM-2 medium (Lonza) supplemented as instructed by the manufacturer. Genomic DNA was extracted with the QIAamp DNA mini kit (Qiagen) and integration sites were cloned by linker-mediated PCR (LM-PCR) as described in (Wu *et al*, 2003). Briefly, 500ng of genomic DNA was digested with MseI and BglII to prevent amplification of internal 5’ LTR fragments. An MseI double-stranded linker was then ligated and ligation-mediated PCR performed with nested primers specific for the linker and the 3’ MoMLV LTR 5’- GACTTGTGGTCTCGCTGTTCCTTGG-3’ and 5’-GGTCTCCTCTGAGTGATTGACTACC-3’. PCR products were shotgun-cloned (TOPO TA cloning kit, Invitrogen) into a library of integration junctions, which was then sequenced. A valid integration contained the MoMLV nested primer, the entire MoMLV genome up to a CA dinucleotide and the linker nested primer. Sequences between the 3’ LTR and the linker primers were mapped onto the human genome by the BLAT genome browser (UCSC Human Genome Project Working Draft, hg19). Sequences featuring a unique best hit with ≥95% identity to the human genome were considered genuine integration sites.

**Supplementary legends**

**Figure S1. Absence of Type COLVII in the patient’s skin and cultured cells.**

**A.** Skin samples from the RDEB patient and from a normal subject (mastectomy). Histological sections were stained with hematoxyline and eosin; note detachment of epidermis from the dermis in the patient’s skin. COLVII was not detected by immunostaining in RDEB patient’s skin, compared to control (green); Hoechst staining in red (false-coloured). Dotted white line represents dermo-epidermal junction. Scale bar 50μm.

**B**. RDEB cultured keratinocytes and fibroblasts did not express detectable COLVII while it was detected in control keratinocytes (brown). Sections were couterstained with hematoxylin. Scale bar: 50µm

**Figure S2. Design of the recombinant vector.**

Outline of the SIN retroviral vector construct used to correct RDEB epidermal stem cells. A cell line, Flp293A-E1aColVII1, was created to stably produce high titers of retroviral particles bearing the complete *COL7A1* cDNA (118 exons) under the control of a minimal constitutive human promoter (see Schucht *et al*, 2006 ). The construct was designed so that the 5’ LTR of the Moloney murine leukemia virus (MoMLV) had no promoter properties upon integration (SIN construct). RU5: RU5 region from MoMLV LTR, Ψ: packaging signal, pEF1α: human elongation factor 1α minimal promoter, *COL7A1* cDNA: complete cDNA of *COL7A1*, ΔU3: partially deleted U3 enhancer region from MoMLV LTR. SpeI and EcoRV are unique restriction sites used for Southern blotting experiments. SIN: self-inactivating; MoMLV: Moloney murine leukemia virus; LTR: long terminal repeat.

**Figure S3. Performances of RDEB keratinocytes infected with SIN retroviruses bearing a *COL7A1* cDNA.**

The performance of RDEB infected keratinocytes at passage V was evaluated by two criteria: colony forming efficiency and percentage of growing colonies. Conditions were: no infection, no protamine (control); protamine alone (5μg/ml); one overnight infection (single infection) or two overnight infections (double infection) with viral supernatant (MOI 10). 100 cells from each condition were plated in two indicator dishes and grown for 12 days. Cells were fixed and stained with Rhodamine B. The number of colony formed and their shape were used to calculate the colony forming efficiency and the percentage of growing colonies. The infection procedure did not impair the performances of keratinocytes.

**Figure S4. Stable infection efficiency of RDEB keratinocytes with SIN retroviruses bearing a *COL7A1* cDNA.**

RDEB keratinocytes were infected with three different lots of retroviral preparation from the producer clone Flp293A-EIaColVII1. Raw supernatants were obtained from Genethon or prepared in our laboratory (experiment 3). Upper panel: viral lot 1 was used for experiment 1, 4, 5 and 6, viral lot 2 for experiment 2 and viral lot 3 for experiment 3. Cells were double infected as described in Figure S3, and passaged at low density (100 to 500 cells) in 35mm Petri dishes. Cells were grown one week and COLVII was detected by immunocytology. Lower panel: selected pictures of COLVII producing (brown) and non-producing colonies (counterstained blue) one passage after experiment 1. Positive and negative colonies were counted to calculate the infection efficiency. The percentage of infected cells ranged between 29% and 42%.

**Figure S5. Serial transfers of COLVII positive and negative clones isolated from a mass culture.**

Clones were serially passaged once a week up to culture exhaustion. Transduced holoclones (clones 6, 22 and 54) and meroclones (clones 3, 17, 24, 58 and 61) had a similar life span whether expressing COLVII (green) or not (black). H: holoclones; M: meroclones.

**Figure S6. Long-term COLVII expression in corrected stem cells.**

Holoclones were subcultured until no growing colony formed in the indicator dishes (see Figure S5). The last subculture of corrected stem cells was fixed and tested for COLVII expression (DAB, brown). Cells were counterstained with hematoxyline (blue). Clones 6 and 22 still expressed COLVII at passage XVI. Scale bar 50μm.

**Figure S7: Relative telomere length of control and RDEB patient’s cells with passages.**

Relative telomere length was assessed by qPCR for control (YF29 at passage IV and XIV) and RDEB keratinocytes (uncorrected at passage IV and clone 6 at passage XIV). The relative telomere length was maintained during subculture (control passage IV to XIV). We observed a small difference in the relative telomere length in between RDEB keratinocytes and control keratinocytes. We also noticed a small difference between Clone 6 (holoclone) and uncorrected RDEB cells (heterogeneous population of keratinocytes).

**Table S1. Screening of corrected stem cells from RDEB epidermal mass culture infected with SIN retroviruses bearing a *COL7A1* cDNA.**

One hundred and fifty RDEB keratinocytes were cloned from a mass culture double infected (see Figure S4). Sixty-seven clones were obtained from which fifteen were obviously paraclones (*) and thus only fifty-two clones were passaged after a week in culture. A fraction of each clone was then transferred onto indicator dishes (clonal type), and another fraction cultured on coverslips for COLVII immunodetection. Cells from indicator dishes were grown for 12 days, fixed and stained with Rhodamine B. The percentage of aborted colonies was recorded for each clone and the clonal type was determined as described (H: holoclone, M: meroclone, P: paraclone). Presence of COLVII was determined for each clone by immunocytochemistry and scored positive (+) or negative (-). No information was obtained for clones 1, 2, 10 and 14. Nd: not determined. Only clones in blue were further studied.

**Table S2. Proviral integration sites determined by ligation-mediated PCR on transduced epidermal stem cells.**

Genomic DNA from transduced holoclones was extracted and integration sites were cloned by ligation-mediated PCR as described . PCR products were shotgun-cloned into a library of integration junctions, which was then sequenced and mapped onto the human genome. Two integration sites were found in each of the clone 6 and 54. The results for clone 22 were not conclusive due to persistent murine feeder cells contamination. The proviral integration pattern was in favour of random integration as expected. Usefull information about the targetted genes could be highlighted for each proviral integration (refsequence, distance to the next transcription start site and neighbourhood genes).
